# Pain coping strategies and their association with quality of life in people with Parkinson’s disease: A cross-sectional study

**DOI:** 10.1371/journal.pone.0257966

**Published:** 2021-11-01

**Authors:** Tino Prell, Jenny Doris Liebermann, Sarah Mendorf, Thomas Lehmann, Hannah M. Zipprich

**Affiliations:** 1 Department of Neurology, Jena University Hospital, Jena, Germany; 2 Center for Healthy Aging, Jena University Hospital, Jena, Germany; 3 Institute of Medical Statistics, Computer Sciences and Documentation, Jena University Hospital, Jena, Germany; Carl von Ossietzky Universitat Oldenburg, GERMANY

## Abstract

**Objective:**

To develop multidimensional approaches for pain management, this study aimed to understand how PD patients cope with pain.

**Design:**

Cross-sectional, cohort study.

**Setting:**

Monocentric, inpatient, university hospital.

**Participants:**

52 patients with Parkinson’s disease (without dementia) analysed.

**Primary and secondary outcome measures:**

Motor function, nonmotor symptoms, health-related quality of life (QoL), and the Coping Strategies Questionnaire were assessed. Elastic net regularization and multivariate analysis of variance (MANOVA) were used to study the association among coping, clinical parameters, and QoL.

**Results:**

Most patients cope with pain through active cognitive (coping self-statements) and active behavioral strategies (increasing pain behaviors and increasing activity level). Active coping was associated with lower pain rating. Regarding QoL domains, active coping was associated with better physical functioning and better energy, whereas passive coping was associated with poorer emotional well-being. However, as demonstrated by MANOVA, the impact of coping factors (active and passive) on the Short Form 36 domains was negligible after correction for age, motor function, and depression.

**Conclusion:**

Passive coping strategies are the most likely coping response of those with depressive symptoms, whereas active coping strategies are the most likely coping response to influence physical function. Although coping is associated with pain rating, the extent that pain coping responses can impact on QoL seems to be low.

## Introduction

People with Parkinson’s disease (PPD) have various methods for coping or dealing with disease and symptoms [1, 2]. Among others, pain is one of the most bothersome nonmotor problems in PD [3]. Pain is common but often is also underdiagnosed and undertreated in PPD. The etiology and phenotype of pain in PD is complex and often multi-factorial. Pain can be related to PD itself and/or other comorbidities, e.g., due to arthrosis of spine or joints amplified by motor- or non-motor PD symptoms [4–6]. It can occur at any time during the disease course [7]. Pain is often categorized into distinct domains of musculoskeletal, off‐related, nocturnal, orofacial, and radicular pain [8–10]. Pain has significant impact on all aspects of the patient´s life and pain further complicates the ability to manage the motor symptoms [11]. Chronic pain is linked to depression, low self-esteem, frustration and sleep deprivation [12–14].

Independent of pain’s etiology or its response to L-Dopa PPD with pain have to cope with this nonmotor symptom. Successful coping with pain may significantly improve the health-related quality of life (QoL) for patients. However, consensus on the most efficient coping strategy for pain is lacking [15]. The situation is further complicated by a plethora of different methods, theories, and concepts of measuring coping. Generally, coping can be categorized as either active or passive coping [16]. Active coping refers to the patient’s attempt to deal with the pain using internal resources to control the pain. Conversely, passive coping (e.g., praying/hoping) generally refers to the tendency to avoid activity and feeling of helplessness to deal with the pain and relinquish control of the pain to other external resources. Passive coping may lead to physical inactivity, which in turn, is followed by physical deterioration [17]. Also, other categorizations of coping exist. Coping with pain can also be classified into cognitive (e.g., distraction) and behavioral (e.g., taking pain medication) strategies [18].

For example, passive pain coping strategies like catastrophizing and praying and hoping are associated with poorer treatment outcome and determine how destructive nonmotor symptoms can be on the QoL of PPD [19]. Given the high prevalence of pain in PD and its impact on QoL, more data are needed in order to understand coping with pain in PD. This may help to develop multidimensional approaches to treat pain. A multidimensional pain treatment includes pharmacologic and non-pharmacologic methods (e.g., physiotherapy, occupational therapy, behavioral therapy, cognitive-behavioral therapy, stress management, relaxation training) [20, 21].

This exploratory study aimed to answer the following three questions: What is the prevalence of different pain coping strategies? How are different pain coping strategies associated with pain ratings? How do different pain coping styles affect health-related QoL?

## Materials and methods

### Subjects

This observational study recruited consecutively PPD from the ward of the Department of Neurology, Jena University Hospital, Jena, Germany (May 2019 to July 2019). This study was approved by the local ethics committee of the Jena University Hospital (4572-10/15). All subjects gave written informed consent in accordance with the Declaration of Helsinki. All patients came as planned to the hospital and received multimodal treatment by specialized therapists and medication modifications during their stay (German Multimodale Komplexbehandlung bei Morbus Parkinson). Prerequisite for the multimodal complex treatment are the physician expertise for PD, an anti-parkinsonian drug titration as well as the application of activating therapies with a duration of at least 7.5 h per week in a multidisciplinary team (physiotherapists, occupational therapists, and speech therapists, psychologist) [22].

Assessments were conducted at the beginning of the hospital stay (before treatments started). The patients were admitted for the following reasons: an increase in fluctuations, an increase in off phases, evaluation for deep brain stimulation, and worsening of gait and freezing. Only patients without relevant cognitive deficits according to the Montreal Cognitive Assessment (MoCa > 21) were included [23].

### Assessments

The following demographical and PD-related data were collected: age, gender, educational level, marital status, living situation, and professional activity. In addition the following parameters were assessed, because they were found to be related to coping in PD in an earlier study [1]: The Movement Disorder Society-sponsored revision of the Unified Parkinson’s Disease Rating Scale III (MDS-UPDRS III) [24], the revised nonmotor symptoms questionnaire (NMS-Quest) [25], and Hoehn and Yahr staging were used to evaluate motor and nonmotor symptoms; cognition was assessed using the MoCa and depression was assessed using the Beck Depression Inventory II (BDI-II). The Medical Outcomes Study (MOS) Short Form 36 (SF-36) was used to measure health-related QoL [26, 27]. The SF-36 is recommended for people with PD [28] and comprises eight concepts of health: physical functioning, role limitations due to physical problems, pain, general health perceptions, energy/vitality, social functioning, role limitations due to emotional problems, and mental health. Single-dimension scores were calculated according to the predefined standardized scoring algorithms based on the following the instructions given by RAND Health Care (www.rand.org/health-care/surveys_tools/mos/36-item-short-form/scoring.html). Scoring was used according to which items from each scale are summed and rescaled with a standard range from 0 to 100, where a score of 100 denotes the best health.

For pain ratings, the corresponding SF-36 was used: item 21 (How much bodily pain have you had during the past 4 weeks?) and item 22 (During the past 4 weeks, how much did pain interfere with your normal work (including both work outside the home and housework)?); lower scores indicate greater pain.

Coping with pain was assessed using the Coping Strategy Questionnaire (CSQ) (https://igptr.ch/wp-content/uploads/2019/09/CSQ-D.pdf). This is a widely used, validated international questionnaire for pain management strategies which includes both active/passive and cognitive/behavioral coping strategies and perceived efficacy of the used coping strategies [29]. The CSQ was developed by Rosenstiel and Keefe in 1983 and is currently the most widely cited international questionnaire for pain management strategies [30]. The German version, the CSQ-D, was developed in Switzerland in 2006 [29]. The CSQ-D comprises a list with 50 examples of pain management of interviewed patients. The patient must assess what he or she does when in pain and circles the number that applies to him or her. Therefore, the respondent does not necessarily have to be in current pain. The patient assesses each item on a seven-point Likert scale from 0 to 6. The score of a scale is the mean value of the filled items. The score for each scale was transformed into a score from 0 as the worst to 100 as the best: (score/6) × 100. The higher the score, the more significant the factor in the process of coping with pain. The CSQ-D consists of eight scales, each with six items (items 1–48) [29, 31]: The coping strategies diverting attention, reinterpreting pain, coping self‐statements, ignoring pain sensations, and increasing activity level were characterized as active, whereas catastrophizing and praying or hoping were categorized as passive. Items 49 (pain control) and 50 (pain reduction) are scored individually and measure the effectiveness of pain management, less the pain management strategy. The two questions about controlling pain and decreasing pain were categorized as perceived efficacy of the used coping strategies. The CSQ scales were therefore classified into CSQ factors: active and passive pain management strategies and self-efficiency [32].

We used the PRISMA reporting guidelines for the reporting checklist [33].

### Statistics

Statistical analyses were performed using SPSS version 25.0 (IBM, New York, NY, USA) and R version 3.6.2 (R Foundation for Statistical Computing, Vienna, Austria), with a p-value of <0.05 indicating statistical significance. Values are presented as mean and standard deviation and categorical variables are presented as absolute numbers and percentages.

In the first step, using descriptive statistics, the cohort was analyzed, and using the Shapiro–Wilk test, the continuous variables were checked whether they are normally distributed. Group comparisons were performed using ANOVA or Kruskal–Wallis test with Bonferroni correction for continuous values and chi-square or Fisher’s exact test for categorical variables. Using Spearman correlation or Pearson correlation, univariate correlation analyses were performed. Associations between coping and pain ratings or SF-36 domains were studied using elastic net regularization [34]. Elastic net regularization leads to parsimonious models, which are easier to interpret. Variable selection is performed by shrinking parameters toward zero and attenuating overfitting, a well-known problem if regression models are applied with a large number of predictors. Tenfold cross-validation was applied to choose the best model with the lowest mean cross-validated error. Within the elastic net algorithm, variables remain in the model if the prediction error averaged over the ten cross-validation samples is reduced. Regression coefficients of the model with 95% confidence intervals were reported. Elastic net regularization was performed using the package glmnet in R 3.6.2. Post hoc power analysis was performed to determine sufficient sample size for regression analyses [35].

To study the effect of coping factors on the SF-36 domains, multivariate analysis of variance (MANOVA) was used. The dependent variables correlated moderately, and the variance inflation factor of the independent variables was below 10, indicating that multicollinearity was not an issue in the analysis.

#### Patient and public involvement statement

This research was done without patient involvement. Patients were not invited to comment on the study design and were not consulted to develop patient relevant outcomes or interpret the results. Patients were not invited to contribute to the writing or editing of this document for readability or accuracy.

## Results

### Demographic and clinical characteristics

The study sample comprised 20 women and 32 men, aged 74.4 ± 6.6 years. Measures of disease severity indicated moderate motor impairment as assessed using the MDS-UPDRS III (Table 1). Approximately 65.4% (n = 34) of patients were in H&Y stage 3, followed by H&Y stage 2 (n = 8) and stage 4 (n = 8), and two were in stage 1. Table 1 shows the detailed clinical and demographical characteristics.

**Table 1:** Demographic and clinical characteristics.

		n = 52	
General demographics			
Age (mean, SD; years)		74.38	6.60
Sex (n, %)	Female	20	38.5
	Male	32	61.5
Marital status (n, %)	Single	2	3.8
	Married	34	65.4
	Divorced/widowed	13	25.0
	Missing data	3	5.8
Housing situation	Alone	15	28.8
	Not alone	37	71.2
Education (n, %)	Middle school (German Realschule)	12	23.1
	High school (German Abitur, study)	19	36.5
	Lower education level (German Hauptschule, no school)	7	13.5
	Missing data	14	26.9
Professional activity (n, %)	Pensioned	51	98.1
	Part-time employed	1	1.9
Parkinson’s disease characteristics			
Disease duration (mean, SD; years)		8.5	5.21
Hoehn and Yahr stage (median, IQR)		2.93	0.80
MDS-UPDRS III (mean, SD)		31.3	14.6
NMS-Quest (mean, SD)		11.12	4.73
Montreal Cognitive Assessment (mean, SD)		25.13	3.09
Beck Depression Inventory II(mean, SD)		10.85	6.28

Movement Disorder Society-sponsored revision of the Unified Parkinson’s Disease Rating Scale III (MDS-UPDRS III), revised nonmotor symptoms questionnaire (NMS-Quest).

### Pain status

Data about pain were extracted from the corresponding SF-36 pain items. Approximately 39.2% reported none (n = 5), very mild (n = 5), or mild (n = 10) bodily pain, and 60.8% reported moderate (n = 14), severe (n = 14), or very severe (n = 3) pain during the past 4 weeks (one missing SF-36). The pain did interfere with normal work with the following findings: six (11.5%) patients not at all, 10 (19.2%) a little bit, 10 (19.2%) moderately, 19 (36.5%) quite a bit, and six (11.5%) extremely.

### Coping strategies

**Fig 1:**
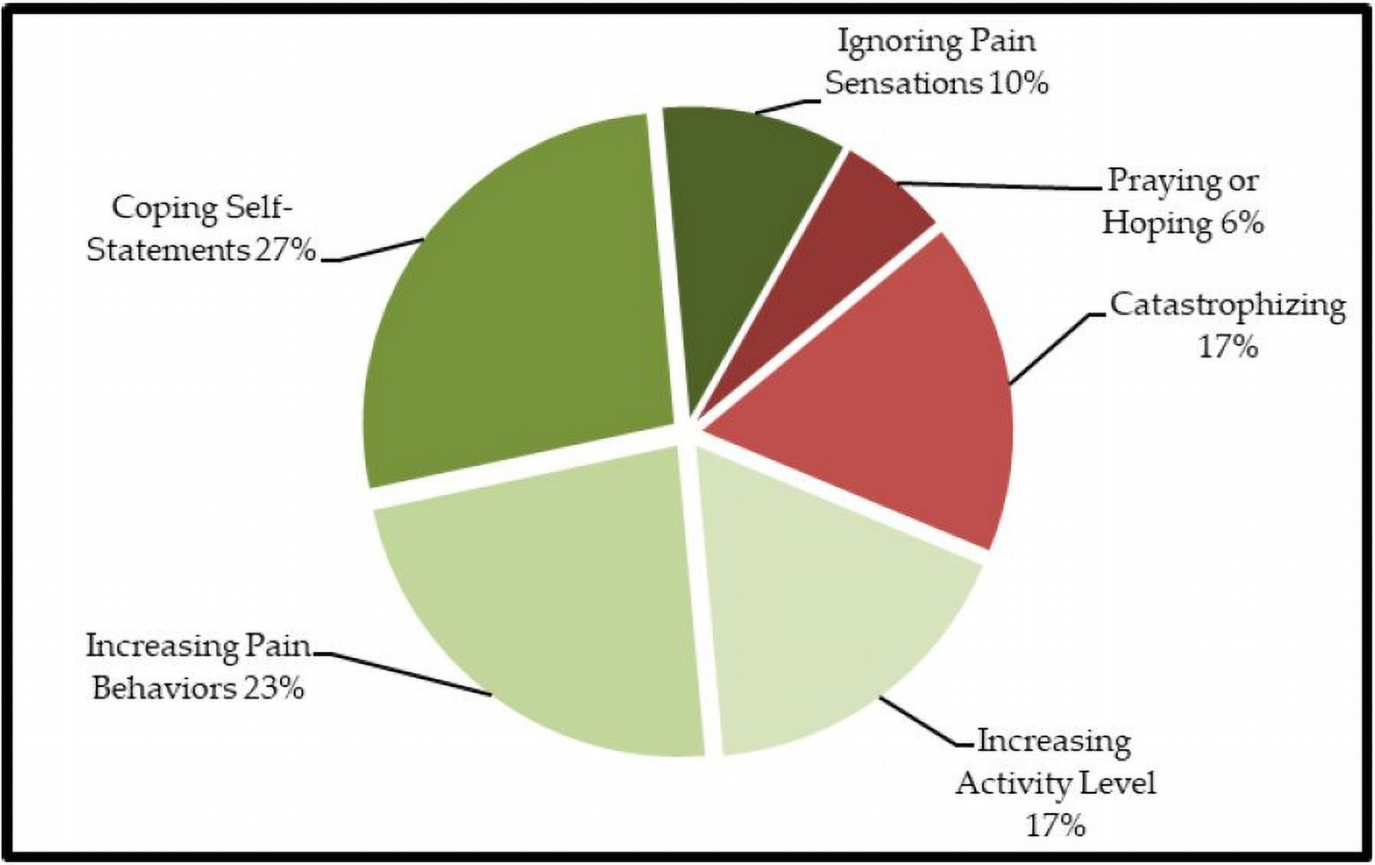
Frequency of Coping Strategy Questionnaire (CSQ) subscales.

Patients with PD usually cope with pain through the active cognitive strategy of coping self-statements and the active behavioral strategies increasing pain behaviors and increasing activity level (Fig 1) (Table 2). The lowest-rated strategy was reinterpreting pain sensations (Table 2). After summarizing the scales to CSQ factors, 33 (63.5%) and 19 (36.5%) used active and passive coping strategies, respectively. Behavioral strategies (n = 38, 73.1%) were more common than cognitive strategies (n = 14, 26.9%).

**Table 2:** Coping Strategy Questionnaire (CSQ)scales in patients with PD (n = 52).

Factor	Strategy	Scale	Mean	SD	Minimum	Maximum
Active	Cognitive	Coping self-statements	52.6	17.0	11.1	83.3
	Cognitive	Ignoring pain sensations	41.5	22.1	0.0	88.9
	Cognitive	Diverting attention	40.4	19.0	0.0	75.0
	Cognitive	Reinterpreting pain sensations	23.0	16.4	0.0	75.0
	Behavioral	Increasing pain behaviors	46.6	15.8	8.3	83.3
	Behavioral	Increasing activity level	43.1	19.3	2.8	83.3
Passive	Cognitive	Catastrophizing	34.8	18.2	0.0	75.0
	Cognitive	Praying or hoping	30.6	16.5	0.0	77.8
Self-efficiency	-	Ability to decrease pain	41.3	26.0	0.0	100.0
	-	Control over pain	42.9	27.0	0.0	100.0

### Perceived effectiveness of the coping strategies

**Fig 2:**
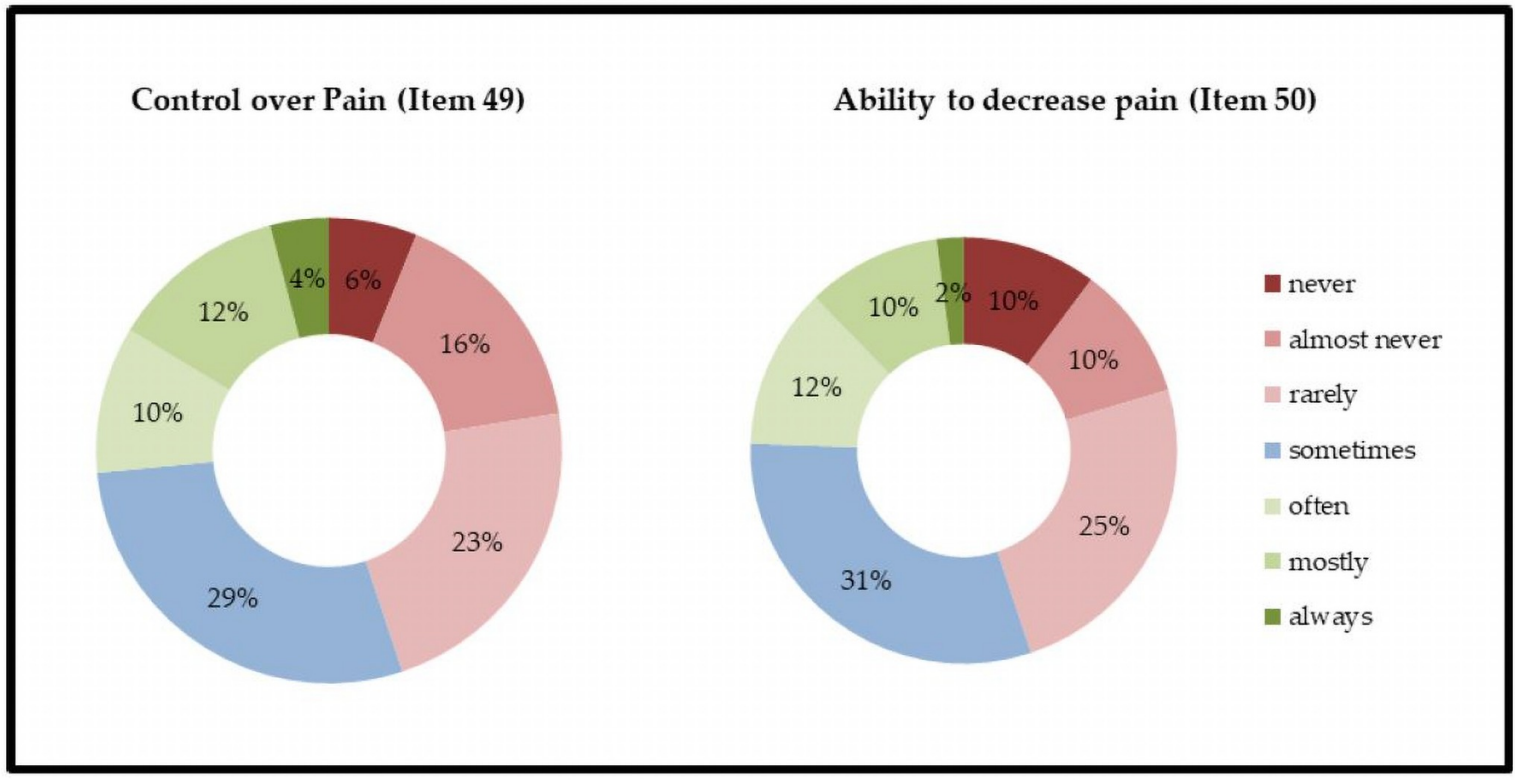
Self-efficiency.

Self-efficiency was assessed using items 49 and 50 (control over pain and ability to decrease pain, respectively). The majority of patients reported the following responses: never, almost never, or rarely able to control or decrease pain (Fig 2). The mean of the factor self-efficiency ((item 49 + 50)/2) was 42.7 (SD = 23.7). Self-efficiency did not differ regarding sex (p = 0.11), living/housing situation (p = 0.29), and educational status (p = 0.58) and did not correlate with age, H&Y, MDS-UPDRS III, NMSQ, MoCa, and BDI (Table 3).

**Table 3:** Spearman correlation between pain Coping Strategy Questionnaire (CSQ) scales and the clinical factors.

Factor	Scale	Age	H&Y	MDS-UPDRS III	NMSQ	MoCa	BDI
Active	Diverting attention	−0.197	−0.245	−0.144	0.273*	−0.074	0.091
	Reinterpreting pain sensations	0.160	−0.419**	−0.235	0.067	−0.197	0.029
	Coping self-statements	0.136	−0.137	−0.043	−0.002	−0.097	−0.070
	Ignoring pain sensations	0.038	−0.203	−0.234	−0.036	−0.014	−0.145
	Increasing activity level	−0.125	−0.300*	−0.262	0.139	0.035	0.024
	Increasing pain behaviors	−0.316*	−0.185	−0.122	0.133	0.105	−0.008
Passive	Praying or hoping	−0.172	−0.203	−0.090	−0.003	−0.174	−0.047
	Catastrophizing	−0.101	−0.052	0.149	0.258	0.018	0.501***
Self-efficiency	Control over pain	0.143	−0.050	0.203	−0.036	−0.090	−0.022
	Ability to decrease pain	−0.007	−0.068	0.058	0.023	0.050	−0.071

Movement Disorder Society-sponsored revision of the Unified Parkinson’s Disease Rating Scale III (MDS-UPDRS III), revised nonmotor symptoms questionnaire (NMS-Quest), Hoehn and Yahr staging (H&Y), Montreal Cognitive Assessment (MoCa), Beck Depression Inventory II (BDI-II).

* p ≤ 0.05,

** p ≤ 0.01,

*** p ≤ 0.001.

### Association between coping and the clinical parameters

In the univariate analyses, some moderate correlations were found between CSQ scales and the clinical parameters (Table 3). The strongest correlation was found between BDI and catastrophizing. The coping scales were not associated with gender, living situation, and educational level (all p > 0.05). Also, the CSQ factors (active and passive) were correlated with some of the clinical parameters. Here active coping was less in higher H&Y stages (r = −0.33, p = 0.02) and passive coping correlated with the BDI (r = 0.32, p = 0.02). The two CSQ factors (active and passive) did not differ regarding sex, marital status, living situation, and educational level (all p < 0.05).

### Health-related QoL

Health-related QoL as measured on the SF36 was substantially impaired in this sample of patients with PD compared with the general adult population. Table 4 shows the mean levels for the eight SF-36 subscales, and for comparison, values reported in the MOS cohort and a PD cohort are shown [36, 37]. The study participants with PD reported poorer health-related QoL on all eight scales of the SF36 compared with the general population and were comparable with the PD cohort by Jenkinson et al. [36].

**Table 4:** Mean levels for the eight SF-36 subscales.

SF-36 domain	Our cohort PD	
	Mean	SD
Physical functioning	39.41	25.01
Role functioning/physical	18.00	29.90
Pain	45.74	25.70
General health	39.22	14.84
Energy/fatigue	44.71	15.34
Social functioning	56.86	27.08
Role functioning/emotional	47.06	44.81
Emotional well-being	60.94	15.79

### Association between coping and pain ratings

Associations between coping factors (active and passive) were analyzed with separate regression on the SF-36 pain subscale (Items 21 and 22) using elastic net regularization. When only the coping factors were entered into the model, active coping was associated with higher SF-36 pain subscale and passive coping with lower SF-36 pain subscale. In a second step, additional clinical and demographic factors were entered into the model (age, gender, H&Y scale, MDS-UPDRS III, MoCa, and BDI-II). In this comprehensive model, lower SF-36 pain subscale (more pain) was associated with female gender, higher H&Y scale, higher MDS-UPDRS III, higher BDI-II, and passive coping. Conversely, active coping was associated with higher SF-36 pain subscale (less pain) (Table 5). In a post hoc power analysis with R^2^ = 0.38 (see Table 5), a statistical power of 0.9, and a significance level of α = 0.05, a sample size of n = 36 would be needed for a significant overall model with 6 predictors [35].

**Table 5:** Elastic net regularization: Predictors of SF-36 pain subscale.

	Coefficient	CI 2.5%	CI 97.5%	t-value	p-value
Gender male	8.48	−4.72	21.68	1.26	0.21
H&Y	−5.10	−13.75	3.56	−1.15	0.25
MDS-UPDRS III	−0.49	−0.94	−0.04	−2.12	0.04
BDI-II	−0.69	−1.73	0.35	−1.30	0.20
Active coping	0.51	0.07	0.95	2.28	0.03
Passive coping	−0.42	−0.91	0.06	−1.72	0.09

MODEL FIT: χ^2^(6) = 12537.61, p < 0.001, Pseudo-R^2^ (Cragg–Uhler) = 0.38, AIC = 466.50, BIC = 481.96.

Note: Movement Disorder Society-sponsored revision of the Unified Parkinson’s Disease Rating Scale III (MDS-UPDRS III), Hoehn and Yahr staging (H&Y), Beck Depression Inventory II (BDI-II).

In the next step, the association between coping styles and SF-36 pain subscale was analyzed. The active strategies Reinterpreting Pain Sensations and Increasing Activity Level were associated with higher SF-36 pain subscale. Catastrophizing and ignoring pain sensations were associated with lower SF-36 pain subscale (Table 6). However, for Ignoring Pain Sensations, the coefficient was quite low and the p-value was high, indicating that this CSQ scale is less important as a predictor for pain rating.

**Table 6:** Elastic net regularization: Association between coping styles and SF-36 pain subscale.

	Coefficient	CI 2.5%	CI 97.5%	t-value	p-value
Reinterpreting pain sensations	0.49	0.02	0.96	2.03	0.05
Ignoring pain sensations	−0.01	−0.48	0.47	−0.02	0.98
Catastrophizing	−0.53	−0.90	−0.17	−2.86	0.01
Increasing activity level	0.27	−0.22	0.76	1.08	0.29

MODEL FIT: χ^2^(4) = 10276.99, p < 0.001, Pseudo-R^2^ (Cragg–Uhler) = 0.31, AIC = 467.84, BIC = 479.43.

### Association between coping and health-related QoL

Some weak to moderate correlations were found between CSQ scales and the SF-36 domains (Table 7, the SF-36 pain domain was not included). Also, coping factors correlated with several SF-36 domains: Active coping was associated with better physical functioning (r = 0.43, p = 0.002) and better energy (r = 0.34, p = 0.02). Passive coping correlated with poorer social functioning (r = −0.30, p = 0.04), poorer role functioning/emotional (r = −0.28, p = 0.045), poorer emotional well-being (r = −0.44, p = 0.001), and poorer energy (r = −0.29, p = 0.04). Self-efficiency correlated with none of the SF-36 domains. Finally, to analyze the impact of coping factors (active and passive) on the SF-36 domains (out of pain scale), a MANOVA was performed. Using a one-way MANOVA, a statistically significant difference was found between coping factors on the combined dependent variables (p = 0.01, partial η^2^ = 0.34, and Wilk’s λ = 0.66). Active or passive coping was associated with physical functioning (p = 0.001, partial η^2^ = 0.23), role functioning/emotional (p = 0.03, partial η^2^ = 0.10), emotional well-being (p = 0.001, partial η^2^ = 0.19), energy (p = 0.001, partial η^2^ = 0.23), and general health (p = 0.03, partial η^2^ = 0.10). However, no significant differences were found between coping factors on the combined dependent variables after entering age, MDS-UPDRS III, and BDI as covariates to the model (p = 0.12, partial η^2^ = 0.24, and Wilk’s λ = 0.75).

**Table 7:** Correlation between CSQ domains and SF-36 domains.

	Diverting attention	Reinterpreting pain sensations	Coping self-statements	Ignoring pain sensations	Increasing activity level	Increasing pain behaviors	Praying or hoping	Catastrophizing
Physical functioning	0.090	0.369**	0.320*	0.443***	0.448***	0.298*	0.011	−0.377**
Social functioning	−0.050	−0.084	0.131	0.218	0.136	0.142	−0.335*	−0.142
Role functioning/physical	−0.135	0.257	0.066	0.179	0.074	−0.085	0.089	−0.176
Role functioning/emotional	−0.032	0.258	0.271	0.406**	0.257	0.022	−0.022	−0.325**
Emotional well-being	−0.157	0.009	0.281	0.322**	0.249	0.024	−0.223	−0.361**
Energy/fatigue	−0.077	0.281	0.421**	0.411**	0.327**	0.208	−0.021	−0.327**
Pain	0.099	0.446***	0.307**	0.446***	0.362**	0.172	0.099	−0.329**
General health	−0.096	0.104	0.210	0.217	0.161	−0.045	0.041	−0.320**

* p ≤ 0.05,

** p ≤ 0.01,

*** p ≤ 0.001.

## Discussion

This exploratory study determined the prevalence of different pain coping strategies in PPD and explored their relationship to pain ratings and health-related QoL. The diagnosis of PD involves long-term physical and emotional consequences for patients, associated with long-term treatment, reduced motor function, and impaired QoL. These factors affect the patient’s mobility, social life, and ability to work. However, the attitude toward the disease and its course, including disease acceptance, may vary considerably from person to person.

Health-related QoL as measured on the SF36 was substantially impaired in this sample of patients with PD compared with the general adult population. The study participants with PD reported poorer health-related QoL on all eight scales of the SF36 compared with the general population and were comparable with the PD cohort by Jenkinson et al. [36]. According to the SF-36 pain items most patients in our cohort experienced any degree of pain during the last 4 weeks before assessment. This is in line with a recent cross-sectional study of German PPD [38]. In general, prevalence rates of pain in PD range from 40 to 85% [39]. This range is caused by differences in terms of study designs and pain questionnaires / assessments. It is therefore important to reflect that we used the SF-36 pain scale rather than more specific PD pain ratings (e.g., King’s PD Pain Questionnaire) as a measure to capture the presence and severity of pain. This was for several reasons: 1) assessment of different PD related and PD non-related pain types was not the primary endpoint of our study, 2) presence of current pain is not necessary to use the CSQ (although the majority of our patients experienced pain), 3) an overwhelming amount of different questionnaires can be–in our experience–difficult in elderly people with PD, 4) other survey-based prevalence studies of pain in PD have already used SF-36 [40, 41]. The SF36 pain scale is a valid and reliable generic questionnaire designed to evaluate bodily pain as a dimension of overall health status. It has been widely used internationally and in diverse populations allowing comparisons across populations. However, the authors acknowledge that for PD other PD specific pain rating scales are available and recommended for clinical trials [42].

We found that pain was associated with female gender, higher H&Y stage, higher motor impairment, depression and passive coping. This is in line with earlier studies reporting that female gender, motor complications, and depression have been found as predictors for pain in PD [5].

Similar to other diseases, this study indicates that PPD choose different types of adaptations or strategies for coping with pain [43]. With self-statements (cognitive strategy) and increasing pain behaviors (behavioral strategy) accounting for 50% of all CSQ styles, the most frequent pain coping strategies in our cohort were active strategies. The most common style, positive coping self-statements or self-instructions, involves the use of self-statements to direct or self-regulate behavior or solve problems. Although they are widely believed to boost mood and self-esteem, their effectiveness is discussed heterogeneously [44]. Our data indicate that positive self-statements are moderately associated with a better physical functioning, better role functioning/emotional, and better energy according to the SF-36. However, positive self-statements were not associated with lower pain rating in the elastic net regularization model. Coping self-statements were also not related to age, gender, or specific PD-related clinical parameters. Generally, this may indicate that this strategy is common but is not necessarily effective in improving pain. However, this cannot be answered conclusively in a cross-sectional design.

The second most used coping style in our cohort was an active and behavioral strategy. The behavioral strategy scales of the CSQ measures (e.g., increasing pain behaviors) overt behaviors aimed at managing pain, including taking medication, resting, walking, and relaxing. Both behavioral CSQ strategies were correlated with better SF-36 physical functioning after controlling for age, depression, and motor function. Moreover, increasing activity level was associated with lower pain rating in the elastic net regularization model. This may indicate that pain behaviors may be efficacious in decreasing the frequency and intensity of pain in this patient group. However, behavioral CSQ strategies, increasing activity level and increasing pain behaviors, were negatively correlated with age and H&Y stage. Accordingly, active coping was less frequently observed in higher H&Y stages. This suggests that behavioral strategies are more common in younger persons and earlier disease stage. Should this observation be confirmed in larger studies, however, this would be dramatic because experiencing pain, as was previously shown, is more common with age and in higher disease stages [45–47]. Conversely, several studies showed that older patients, more often than young adults and middle adulthood, declared that they cope with pain in a more active manner [44, 48]. Thus, for future studies, at this point elucidating whether behavioral strategies are actually used less frequently by older patients would be interesting, and if so, where do the obstacles lie.

Passive strategies (praying or hoping and catastrophizing) were negatively correlated with pain rating and the SF-36, although the correlations with the SF-36 domains did not reach statistical significance after correction for BDI-II. This is consistent with the general observation that active coping is generally associated with more adaptive adjustment [16, 49].

Hence, individual associations between individual CSQ scales and individual non-pain SF-36 domains are present. However, if the CSQ scales are combined with active and passive factors, these associations between pain coping and QoL must be at least partially relativized. Given the strength of the correlations and the results of the MANOVA, active or passive coping must be stated to only have a small negligible influence on general QoL. The strongest association is understandably between coping and the SF-36 pain domain/pain ratings. Moreover, active coping can only be cautiously stated to be associated with better physical functioning and passive coping with depression or poor emotional well-being. The fact that pain coping is only slightly associated with health-related QoL is surprising and needs further consideration. The SF-36 is a generic instrument, and the domain physical functioning mainly covers aspects related to mobility (walking, carrying, bending, kneeling, etc.). These mobility aspects are usually impaired during disease course in PD. It seems that other factors, including MDS-UPDRS III or H&Y stage, play a more essential role for the SF-36 domains in our PPD, i.e., the way of dealing with pain.

Several limitations in this study should be noted. The CSQ asks how people would cope with pain if they were in pain. Therefore, the CSQ is not limited to the use of patients who are currently in pain. Nevertheless, it should be noted that a small proportion of patients reported (n = 5) that they had not experienced pain in the 4 weeks before assessment. The authors acknowledge that a small group of patients, one coping evaluating tool, and one pain measurement limit the research. This was a preliminary and explorative study. The study sample included PPD without severe cognitive impairment treated at a specialty movement disorders clinic, thus generalizing our findings to those with cognitive impairment and community-dwelling people with PD is difficult. In addition, the participants in this study rather reflect people who have more advanced PD. Because the data from this cross-sectional study are correlational, drawing causal conclusions from the results is not possible. These causal relationships should be tested in future longitudinal studies. QoL is influenced by both external and internal factors. Controlling all possible external factors (financial situation, social contacts, treatment, etc.) was not possible while assessing important external factors such as educational level and marital status. Furthermore, other internal resources or personality traits are relevant when studying causal relations between coping and QoL. Regarding PD, the authors acknowledge that nonmotor symptoms were only roughly assessed using a comprehensive screening tool. Moreover, we cannot make a statement about the interaction between medical, antalgic treatment and coping with the current data.

## Conclusions

Given the results and limitations of this study, some conclusions can be drawn. The present findings point to passive strategies as the most likely response of those with depressive symptoms and active strategies as the most likely coping response to influence physical function. Therefore, further exploration of how coping strategies may change pain perception over time is warranted. Assessing patient’s coping with pain as part of multidisciplinary pain treatment may also be useful to identify patients at the highest risk of poor adjustment to pain. Although pain is common in PD, the extent that pain coping responses impact on overall QoL seems to be low. Against the background of the heterogeneity of PD, the association between age, disease stage, and coping with pain should be further investigated in future studies.

## Supporting information

S1 Checklist STROBE statement—Checklist of items that should be included in reports of *cohort studies*. (DOC)

## References

[1] Liebermann JD, Witte OW, Prell T. Association between different coping styles and health-related quality of life in people with Parkinson’s disease: a cross-sectional study. BMJ Open. 2020;10(7):e036870. doi: 10.1136/bmjopen-2020-036870 32665390 PMC7365430

[2] Nilsson MH, Oswald F, Palmqvist S, Slaug B. Coping Styles among People with Parkinson’s Disease: A Three-Year Follow-Up Study. Behav Sci (Basel). 2020;10(12). doi: 10.3390/bs10120190 33322716 PMC7763158

[3] Uebelacker LA, Epstein-Lubow G, Lewis T, Broughton MK, Friedman JH. A survey of Parkinson’s disease patients: most bothersome symptoms and coping preferences. J Parkinsons Dis. 2014;4(4):717–23. doi: 10.3233/JPD-140446 25271239

[4] Buhmann C, Wrobel N, Grashorn W, Fruendt O, Wesemann K, Diedrich S, et al. Pain in Parkinson disease: a cross-sectional survey of its prevalence, specifics, and therapy. J Neurol. 2017;264(4):758–69. doi: 10.1007/s00415-017-8426-y 28243753

[5] Buhmann C, Ip CW, Oehlwein C, Tönges L, Wolz M, Reichmann H, et al. [Parkinson Disease and Pain—diagnostic and therapeutic approaches to a challenging non-motor symptom]. Fortschr Neurol Psychiatr. 2018;86(S 01):S48–s58. 30016807 10.1055/a-0590-4464

[6] Buhmann C, Kassubek J, Jost WH. Management of Pain in Parkinson’s Disease. J Parkinsons Dis. 2020;10(s1):S37–s48. doi: 10.3233/JPD-202069 32568113 PMC7592654

[7] Antonini A, Tinazzi M, Abbruzzese G, Berardelli A, Chaudhuri KR, Defazio G, et al. Pain in Parkinson’s disease: facts and uncertainties. Eur J Neurol. 2018;25(7):917–e69. doi: 10.1111/ene.13624 29520899

[8] Chaudhuri KR, Rizos A, Trenkwalder C, Rascol O, Pal S, Martino D, et al. King’s Parkinson’s disease pain scale, the first scale for pain in PD: An international validation. Movement disorders: official journal of the Movement Disorder Society. 2015;30(12):1623–31. doi: 10.1002/mds.26270 26096067

[9] Ford B. Pain in Parkinson’s disease. Clin Neurosci. 1998;5(2):63–72. 10785830

[10] Ford B. Pain in Parkinson’s disease. Movement disorders: official journal of the Movement Disorder Society. 2010;25 Suppl 1:S98–103. doi: 10.1002/mds.22716 20187254

[11] Twomey D, Stuart S, Baker K. Pain in Parkinson’s disease: The lived experience. International Journal of Therapy and Rehabilitation. 2018;25.

[12] Rana AQ, Qureshi ARM, Haris A, Danish MA, Furqan MS, Shaikh O, et al. Negative impact of severity of pain on mood, social life and general activity in Parkinson’s disease. Neurological Research. 2018;40(12):1054–9. doi: 10.1080/01616412.2018.1517852 30221591

[13] Ehrt U, Larsen JP, Aarsland D. Pain and its relationship to depression in Parkinson disease. Am J Geriatr Psychiatry. 2009;17(4):269–75. doi: 10.1097/jgp.0b013e31818af7ef 19322934

[14] Nègre-Pagès L, Regragui W, Bouhassira D, Grandjean H, Rascol O. Chronic pain in Parkinson’s disease: the cross-sectional French DoPaMiP survey. Movement disorders: official journal of the Movement Disorder Society. 2008;23(10):1361–9. doi: 10.1002/mds.22142 18546344

[15] Baastrup S, Schultz R, Brodsgaard I, Moore R, Jensen TS, Vase Toft L, et al. A comparison of coping strategies in patients with fibromyalgia, chronic neuropathic pain, and pain-free controls. Scand J Psychol. 2016;57(6):516–22. doi: 10.1111/sjop.12325 27558974

[16] Brown GK, Nicassio PM. Development of a questionnaire for the assessment of active and passive coping strategies in chronic pain patients. Pain. 1987;31(1):53–64. doi: 10.1016/0304-3959(87)90006-6 3696743

[17] Mellegård M, Grossi G, Soares JJF. A comparative study of coping among women with fibromyalgia, neck/shoulder and back pain. International Journal of Behavioral Medicine. 2001;8(2):103–15.

[18] Fernandez E. A classification system of cognitive coping strategies for pain. Pain. 1986;26(2):141–51. doi: 10.1016/0304-3959(86)90070-9 3531980

[19] Lerman SF, Bronner G, Cohen OS, Elincx-Benizri S, Strauss H, Yahalom G, et al. Catastrophizing mediates the relationship between non-motor symptoms and quality of life in Parkinson’s disease. Disabil Health J. 2019;12(4):673–8. doi: 10.1016/j.dhjo.2019.03.006 30928237

[20] Liu WC, Zheng ZX, Tan KH, Meredith GJ. Multidimensional Treatment of Cancer Pain. Curr Oncol Rep. 2017;19(2):10. doi: 10.1007/s11912-017-0570-0 28220448

[21] Skogar O, Lokk J. Pain management in patients with Parkinson’s disease: challenges and solutions. J Multidiscip Healthc. 2016;9:469–79. doi: 10.2147/JMDH.S105857 27757037 PMC5053370

[22] Richter D, Bartig D, Muhlack S, Hartelt E, Scherbaum R, Katsanos AH, et al. Dynamics of Parkinson’s Disease Multimodal Complex Treatment in Germany from 2010(-)2016: Patient Characteristics, Access to Treatment, and Formation of Regional Centers. Cells. 2019;8(2). 10.3390/cells8020151 PMC6406830 30754730

[23] Nasreddine ZS, Phillips NA, Bédirian V, Charbonneau S, Whitehead V, Collin I, et al. The Montreal Cognitive Assessment, MoCA: a brief screening tool for mild cognitive impairment. J Am Geriatr Soc. 2005;53(4):695–9. doi: 10.1111/j.1532-5415.2005.53221.x 15817019

[24] Goetz CG, Fahn S, Martinez-Martin P, Poewe W, Sampaio C, Stebbins GT, et al. Movement Disorder Society-sponsored revision of the Unified Parkinson’s Disease Rating Scale (MDS-UPDRS): Process, format, and clinimetric testing plan. Movement disorders: official journal of the Movement Disorder Society. 2007;22(1):41–7. 17115387 10.1002/mds.21198

[25] Romenets SR, Wolfson C, Galatas C, Pelletier A, Altman R, Wadup L, et al. Validation of the non-motor symptoms questionnaire (NMS-Quest). Parkinsonism Relat Disord. 2012;18(1):54–8. doi: 10.1016/j.parkreldis.2011.08.013 21917501

[26] Ware JE Jr, Sherbourne CD. The MOS 36-item short-form health survey (SF-36). I. Conceptual framework and item selection. Med Care. 1992;30(6):473–83. 1593914

[27] McHorney CA, Ware JE Jr, Lu JF, Sherbourne CD. The MOS 36-item Short-Form Health Survey (SF-36): III. Tests of data quality, scaling assumptions, and reliability across diverse patient groups. Med Care. 1994;32(1):40–66. doi: 10.1097/00005650-199401000-00004 8277801

[28] Martinez-Martin P, Jeukens-Visser M, Lyons KE, Rodriguez-Blazquez C, Selai C, Siderowf A, et al. Health-related quality-of-life scales in Parkinson’s disease: critique and recommendations. Mov Disord. 2011;26(13):2371–80. doi: 10.1002/mds.23834 21735480

[29] Verra ML, Angst F, Lehmann S, Aeschlimann A. Translation, cross-cultural adaptation, reliability, and validity of the German version of the Coping Strategies Questionnaire (CSQ-D). J Pain. 2006;7(5):327–36. doi: 10.1016/j.jpain.2005.12.005 16632322

[30] Rosenstiel AK, Keefe FJ. The use of coping strategies in chronic low back pain patients: relationship to patient characteristics and current adjustment. Pain. 1983;17(1):33–44. doi: 10.1016/0304-3959(83)90125-2 6226916

[31] Verra M. Die Schmerzbewältigung erfassen. physiopraxis. 2007;5(07/08):40–1.

[32] Tan G, Jensen MP, Robinson-Whelen S, Thornby JI, Monga TN. Coping with chronic pain: a comparison of two measures. Pain. 2001;90(1–2):127–33. doi: 10.1016/s0304-3959(00)00395-x 11166978

[33] Moher D, A L, J T, DG A. Preferred Reporting Items for Systematic Reviews and Meta-Analyses: The PRISMA Statement. In: Group TP, editor. PMC3090117 21603045

[34] Zou H, Hastie T. Regularization and Variable Selection via the Elastic Net. Journal of the Royal Statistical Society Series B (Statistical Methodology). 2005;67(2):301–20.

[35] Cohen J. Statistical power analysis for the behavioral sciences. Hillsdale, N.J.: L. Erlbaum Associates; 1988.

[36] Jenkinson C, Peto V, Fitzpatrick R, Greenhall R, Hyman N. Self-reported functioning and well-being in patients with Parkinson’s disease: comparison of the short-form health survey (SF-36) and the Parkinson’s Disease Questionnaire (PDQ-39). Age Ageing. 1995;24(6):505–9. doi: 10.1093/ageing/24.6.505 8588541

[37] Hays RD, Sherbourne CD, Mazel R. User’s Manual for the Medical Outcomes Study (MOS) Core Measures of Health-Related Quality of Life: RAND Corporation; 1995.

[38] Buhmann C, Wrobel N, Grashorn W, Fruendt O, Wesemann K, Diedrich S, et al. Pain in Parkinson disease: a cross-sectional survey of its prevalence, specifics, and therapy. Journal of neurology. 2017;264(4):758–69. doi: 10.1007/s00415-017-8426-y 28243753

[39] Broen MP, Braaksma MM, Patijn J, Weber WE. Prevalence of pain in Parkinson’s disease: a systematic review using the modified QUADAS tool. Movement disorders: official journal of the Movement Disorder Society. 2012;27(4):480–4. doi: 10.1002/mds.24054 22231908

[40] Wasner G, Deuschl G. Chapter 50 Pain in Parkinson’s disease. Handb Clin Neurol. 2006;81:747–60. doi: 10.1016/S0072-9752(06)80054-0 18808872

[41] Beiske AG, Loge JH, Rønningen A, Svensson E. Pain in Parkinson’s disease: Prevalence and characteristics. Pain. 2009;141(1–2):173–7. doi: 10.1016/j.pain.2008.12.004 19100686

[42] Perez-Lloret S, Ciampi de Andrade D, Lyons KE, Rodríguez-Blázquez C, Chaudhuri KR, Deuschl G, et al. Rating Scales for Pain in Parkinson’s Disease: Critique and Recommendations. Movement disorders clinical practice. 2016;3(6):527–37. doi: 10.1002/mdc3.12384 30363588 PMC6178703

[43] Religioni U, Czerw A, Badowska-Kozakiewicz AM, Deptała A. Assessment of Pain, Acceptance of Illness, Adjustment to Life, and Strategies of Coping with Illness among Patients with Gastric Cancer. Journal of Cancer Education. 2020;35(4):724–30. doi: 10.1007/s13187-019-01519-0 30972579 PMC7363718

[44] Wood JV, Perunovic WQ, Lee JW. Positive self-statements: power for some, peril for others. Psychol Sci. 2009;20(7):860–6. doi: 10.1111/j.1467-9280.2009.02370.x 19493324

[45] Buchner M, Neubauer E, Zahlten-Hinguranage A, Schiltenwolf M. Age as a predicting factor in the therapy outcome of multidisciplinary treatment of patients with chronic low back pain—a prospective longitudinal clinical study in 405 patients. Clin Rheumatol. 2007;26(3):385–92. doi: 10.1007/s10067-006-0368-1 16865309

[46] Verra ML, Angst F, Staal JB, Brioschi R, Lehmann S, Aeschlimann A, et al. Reliability of the Multidimensional Pain Inventory and stability of the MPI classification system in chronic back pain. BMC Musculoskelet Disord. 2012;13:155. doi: 10.1186/1471-2474-13-155 22916687 PMC3495019

[47] Lansbury G. Chronic pain management: a qualitative study of elderly people’s preferred coping strategies and barriers to management. Disabil Rehabil. 2000;22(1–2):2–14. doi: 10.1080/096382800297079-1 10661753

[48] Baker TA, Green CR. Intrarace differences among black and white americans presenting for chronic pain management: the influence of age, physical health, and psychosocial factors. Pain medicine (Malden, Mass). 2005;6(1):29–38. 10.1111/j.1526-4637.2005.05014.x 15669948

[49] Nicholas MK, Wilson PH, Goyen J. Comparison of cognitive-behavioral group treatment and an alternative non-psychological treatment for chronic low back pain. Pain. 1992;48(3):339–47. doi: 10.1016/0304-3959(92)90082-M 1534400

